# Bibliometric analysis: Hot spots and frontiers in acupuncture treatment of cerebral infarction

**DOI:** 10.1097/MD.0000000000037800

**Published:** 2024-04-12

**Authors:** Yutong Han, Chang Liu, Xinming Yang, Jiaxiao Zhou, Weiping Shi, Huasong Gao, Huixue Zhang, Dawei Ran, Lei Shi

**Affiliations:** a Department of Acupuncture, First Teaching Hospital of Tianjin University of Traditional Chinese Medicine, National Clinical Research Center for Chinese Medicine Acupuncture and Moxibustion, Tianjin, China; b Guang’anmen Hospital, China Academy of Chinese Medical Sciences, Beijing, China.

**Keywords:** acupuncture, bibliometric analysis, cerebral infarction

## Abstract

**Objective:**

CiteSpace6.1.R2 is used to analyze the research status of acupuncture in the treatment of cerebral infarction, and to find relevant hot spots and frontiers.

**Methods:**

The researchers searched the Web of Science Core Collection database. The search date is from the establishment of the database to August 31, 2023. The search terms and expressions are: (“Cerebral Infarction” OR “Ischemic stroke”) AND (“Acupuncture” OR “fire needle”). The researchers used CiteSpace software to draw a knowledge map to explore the hot spots and frontiers of acupuncture in treating cerebral infarction.

**Results:**

We screened 414 articles in the Web of Science Core Collection database. China is the country with the largest number of publications, with a total of 343 papers published. China’s institutions cooperate most closely, and cooperation between countries is less and more scattered. The author with the highest number of published articles is Chen L, with a total of 31 published articles. The research focus mainly revolves around the mechanism of acupuncture treatment of cerebral infarction and electroacupuncture treatment of cerebral infarction. Among them, acupuncture treatment of cerebral infarction is the most.

**Conclusion:**

According to CiteSpace’s analysis results, China is at the forefront of this research field, while other countries have less research in this field and little cooperation among countries. At present, the mainstream aspect of research is the mechanism of acupuncture treatment of cerebral infarction electroacupuncture and acupuncture points. Therefore, in future research, we should pay more attention to the treatment of cerebral infarction mechanism of acupuncture, problems with the type of acupuncture used, and acupuncture points.

## 1. Introduction

Stroke is by far the second leading cause of death rate and disability in the world, putting enormous pressure on all aspects of social development.^[1]^ The most common type of stroke is ischemic stroke (cerebral infarction), which accounts for 70% to 80% of strokes.^[2]^ A series of clinical randomized controlled experimental studies on acupuncture in the treatment of cerebral infarction shows that acupuncture is not only widely used in the study of cerebral infarction, but also has good therapeutic effect and high safety.^[3–5]^ Acupuncture has been listed by the World Health Organization as an alternative and complementary therapy for the treatment and improvement of cerebral infarction.^[6–8]^ However, so far, few studies have used scientific bibliometric methods to conduct comprehensive statistical analysis in the field of acupuncture treatment of cerebral infarction. Therefore, through the literature measurement and knowledge map visualization analysis of acupuncture therapy for cerebral infarction in the Web of Science Core Collection (WoSCC) database, aiming to explore the research status and future development trend of acupuncture in the field of treating cerebral infarction at home and abroad, provide research ideas for scholars.

CiteSpace can visualize the structure, laws, and distribution of scientific knowledge to form a map of scientific knowledge.^[9–11]^ Accordingly, CiteSpace6.1 is proposed for the present study. The R2 software is utilized for an analysis of the literature on acupuncture treatment of cerebral infarction in the WoSCC database. This is the first visual analysis of CiteSpace in the field of acupuncture for the treatment of cerebral infarction. In order to objectively reveal the basic situation in the field of acupuncture treatment of cerebral infarction, research hotspots, and frontiers.

## 2. Methods

### 2.1. Data sources and search policies

**Figure 1.**
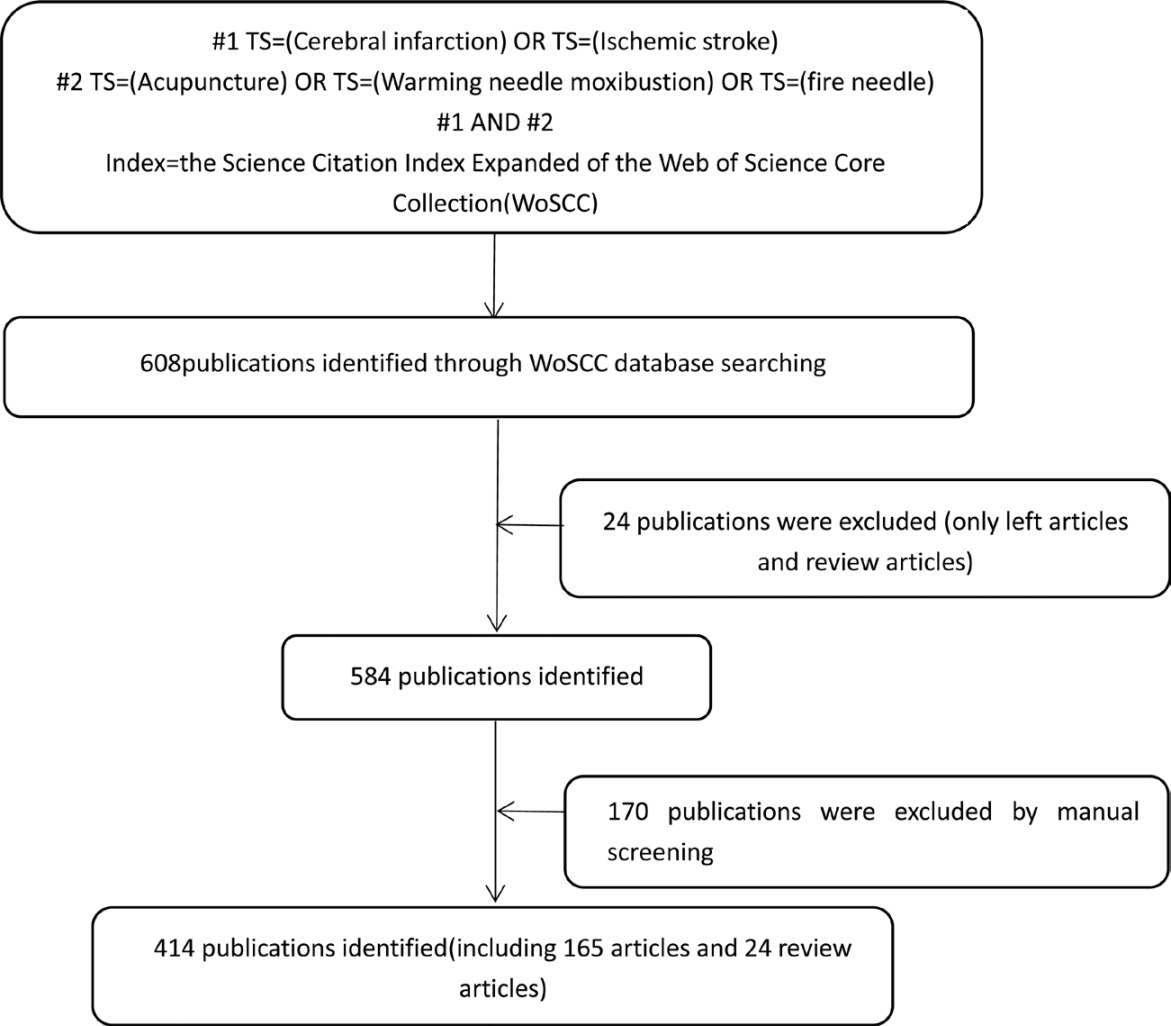
Flowchart of literature screening. WoSCC = Web of Science Core Collection database.

As shown in Figure 1, in order to avoid the impact of database article updates on the search. The researchers completed the search of the WoSCC database on August 18, 2022. The search terms were: (“Cerebral Infarction” OR “Ischemic stroke”) AND (“Acupuncture” OR “Warming Needle Moxibustion” OR “fire needle”) and yielded 414 results. The search date is from the establishment of the library to August 18, 2022.

### 2.2. Patient and public involvement

Patients or the public were not involved in the design, conduct, reporting, or dissemination plans of our research.

### 2.3. Inclusion criteria

The researchers only included articles and reviews of type in the WoSCC database, and 2 researchers checked the articles titled abstracts to screen out articles unrelated to the research content. Finally, the researchers screened out 414 articles for visual analysis.

### 2.4. Bibliometric analysis tools—CiteSpace

CiteSpace is a bibliometric software for visual analysis of scientific knowledge graphs. We produce relevant statistical charts and tables in Microsoft Word and Excel based on the analysis results of CiteSpace. Additionally, we conduct the country through CiteSpace, agencies, authors, and keywords. The analysis of the map cited in the literature can judge the research hotspots and frontiers in the field of acupuncture treatment of cerebral infarction.

## 3. Results

### 3.1. Quantitative analysis of the publication year

**Figure 2.**
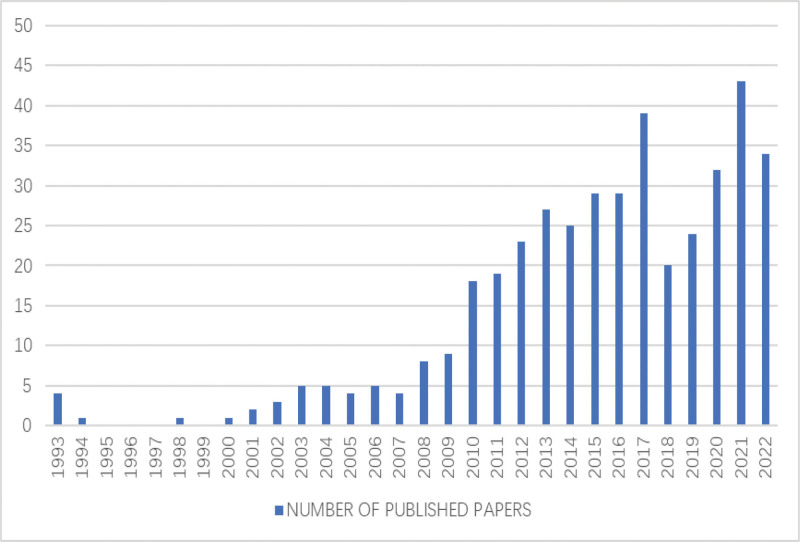
Annual trend chart for publications.

Based on the analysis of 414 articles finally included in the WoSCC database. Between 1993 and 2022, trends in acupuncture treatment of cerebral infarction have fluctuated. However, the overall results showed steady growth. As shown in Figure 2, the research became colder in 2018, but after 2018, the research heat of acupuncture for cerebral infarction gradually warmed up, and reached a new peak in 2021.

### 3.2. Analysis of the cooperation map of countries and institutions

**Figure 3.**
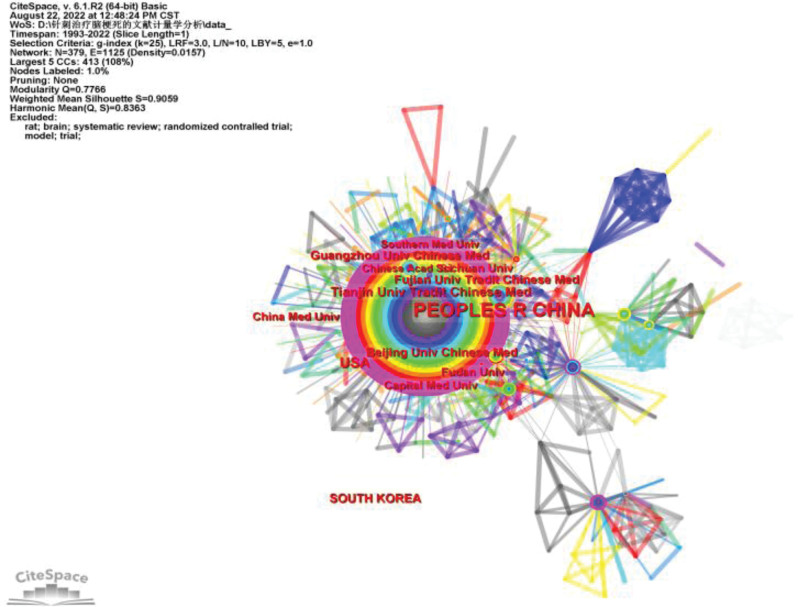
National and institutional network diagram.

In the cooperation map of countries and institutions, the larger the node, the more times the country or institution appears and the more documents are issued. The connection between nodes indicates the degree of close cooperation between countries or institutions. Mediation centrality is an important index to measure the importance of nodes in the network, so as to find and measure the importance of nodes in the network. The higher the centrality of intermediaries, the greater the influence of nodes in the cooperative network of countries and institutions. A total of 147 papers were published by the top 5 institutions and 408 papers were published by the top 5 countries (Table 1). Among them, the cooperation between various institutions in China is the closest, and the cooperation between various countries is less and more scattered. The outer part of the corresponding node of Peoples R China is wrapped in a purple circle, indicating that Peoples R China has a high center of intermediary, that is, China has a strong influence in this research field (Fig. 3).

**Table 1:** Top 5 countries and agencies.

Rank	Institution	N (%)	Country	N (%)
1	Tianjin Univ Tradit Chinese Med	42 (10.14)	Peoples R China	343 (82.85)
2	Fujian Univ Tradit Chinese Med	31 (7.49)	South Korea	29 (6.52)
3	Capital Med Univ	27 (6.52)	USA	27 (6.52)
4	Guangzhou Univ Chinese Med	24 (5.80)	Germany	5 (1.21)
5	Fudan Univ	23 (5.56)	England	4 (0.97)

### 3.3. The authors present a collaborative network analysis

**Figure 4.**
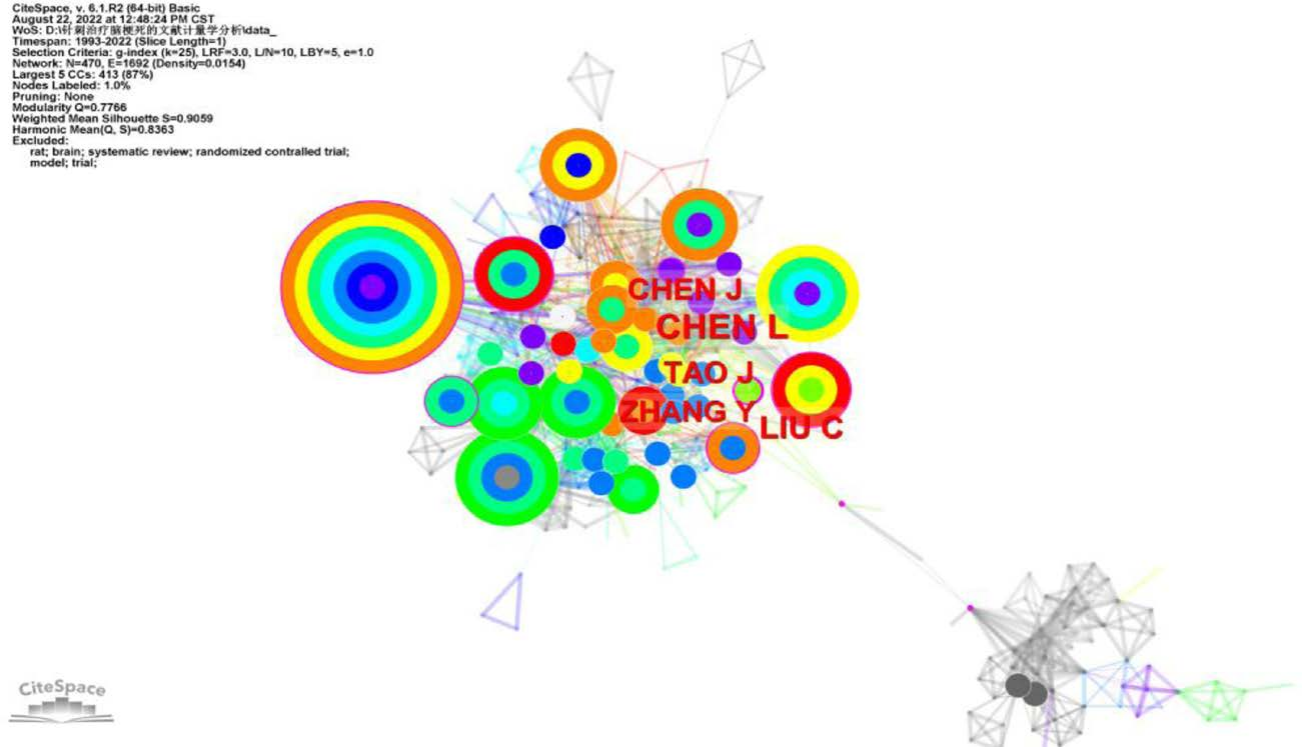
Author collaboration network diagram.

Each node in the author cooperation map corresponds to 1 author, and the larger the node, the larger the number of documents issued by the author. The connection between nodes indicates the strength of cooperation between authors, and the thicker the connection, the closer the cooperation. The result shows that the number of nodes N = 470, the number of connections *E* = 1692, and the module value is modularity (*Q*) = 0.7766. The average contour value means Silhouette (*S*) = 0.9059 indicating that a total of 470 authors appear. There were 1692 collaborations between authors. Among them, by Chen L, Liu C, and Chen J, the author cooperation network represented by Tao J is large the higher volume of posts. But with other research teams (with Cao C, Ya-Ping L, Han Y, Shen Y, and Yang Z represent less cooperation between each other. The top 10 authors have published a total of 180 papers (43.48%) (Table 2). These include Chen L, Wang J, Wang L, Wang Y, Li Y, and Yang S. The outside of the corresponding node of Zhang B is wrapped in a purple circle. This indicates that the mediating centrality of these authors is high. They are in different communities of authors, with strong information control and influence (Fig. 4).

**Table 2:** The top 10 authors.

Rank	Author	Count of articles	Year of first article
1	Chen L	31	2012
2	Liu C	23	2006
3	Chen J	20	2003
4	Tao J	20	2013
5	Zhang Y	17	2014
6	Wang J	15	2008
7	Wang Y	14	2015
8	Li X	14	2012
9	Yang J	13	2014
10	Li J	13	2008

### 3.4. Keyword analysis

**Figure 5.**
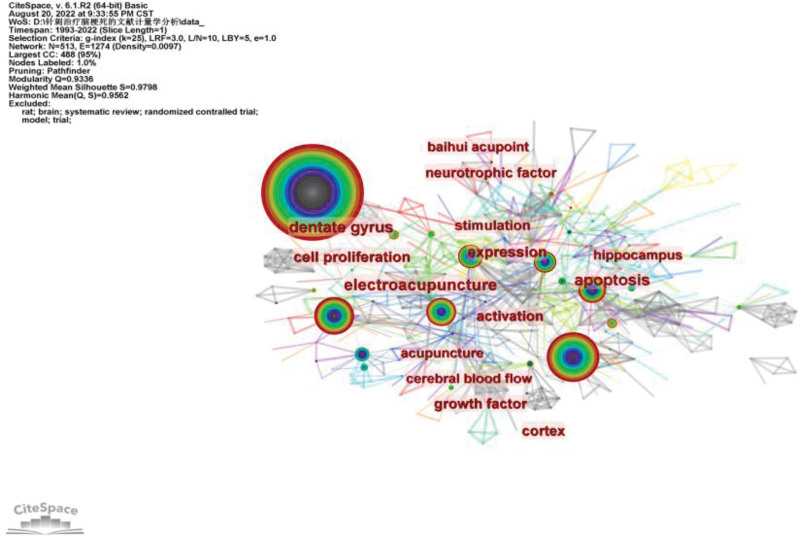
Keyword network diagram.

High-frequency keywords can be regarded as hot topics in this field of research, and high-center keywords can be regarded as topics with high influence in this field. After analysis, the top 10 high-center keywords are: electroacupuncture (centrality: 0.25), neurotrophic factor (centrality: 0.21), apoptosis (centrality: 0.19), dentate gyrus (centrality: 0.19), activation (centrality: 0.18), Ischemic stroke (centrality: 0.17), Baihui acupoint (centrality: 0.17), expression (centrality: 0.15), stimulation (centrality: 0.14), cell proliferation (centrality: 0.14). Top 10 high-frequency keywords are: Ischemic stroke (frequency: 305), acupuncture (frequency: 128), recovery (frequency: 79), electroacupuncture (frequency: 56), expression (frequency: 53), stimulation (frequency: 52), activation (frequency: 49), neural regeneration (frequency: 30), apoptosis (frequency: 26), mechanism (frequency: 26) (Table 3). High centrality keywords and high-frequency keywords are basically the same (Fig. 5).

**Table 3:** Top 10 keywords.

Rank	Count	Keywords	Centrality	Keywords
1	305	Ischemic stroke	0.25	electroacupuncture
2	128	acupuncture	0.21	neurotrophic factor
3	79	recovery	0.19	apoptosis
4	56	electroacupuncture	0.19	dentate gyrus
5	53	expression	0.18	activation
6	52	stimulation	0.17	Ischemic stroke
7	49	activation	0.17	Baihui acupoint
8	30	neural regeneration	0.15	expression
9	26	apoptosis	0.14	stimulation
10	22	mechanism	0.13	cell proliferation

### 3.5. Analysis of keyword cluster atlas

**Figure 6.**
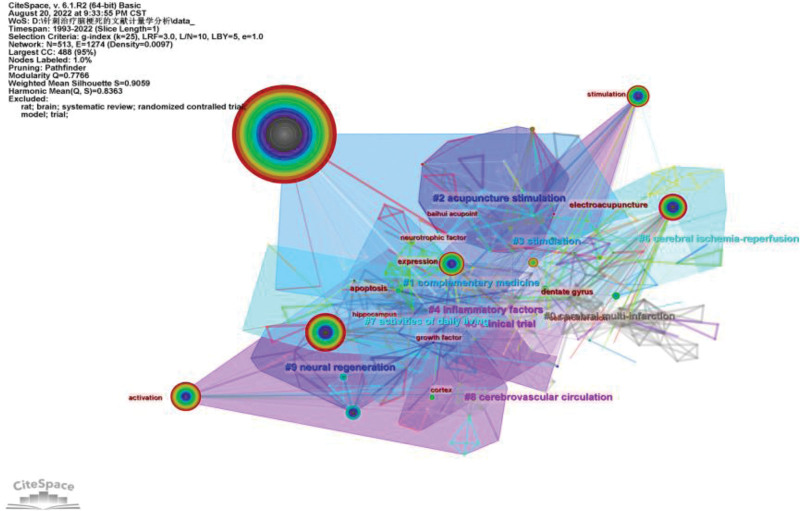
Keyword clustering diagram.

Keyword map generated a total of 34 clusters, of which the top 10 clustering. Table 4 shows that the size is >10, the clustering effect is better, Silhouette is >0.8, and the tightness of each group member is good (Fig. 6).

**Table 4:** Top 9 keyword clusters.

Cluster ID	Size	Top terms (log-likelihood ratio, p-level)	Silhouette
#0	56	Ischemic stroke	0.841
#1	40	acupuncture	0.908
#2	39	recovery	0.925
#3	39	Electroacupuncture	0.884
#4	34	expression	0.918
#5	33	stimulation	0.919
#6	33	activation	0.874
#7	29	neural regeneration	0.824
#8	27	apoptosis	0.96
#9	27	mechanism	0.906

### 3.6. Keyword outburst analysis

**Figure 7.**
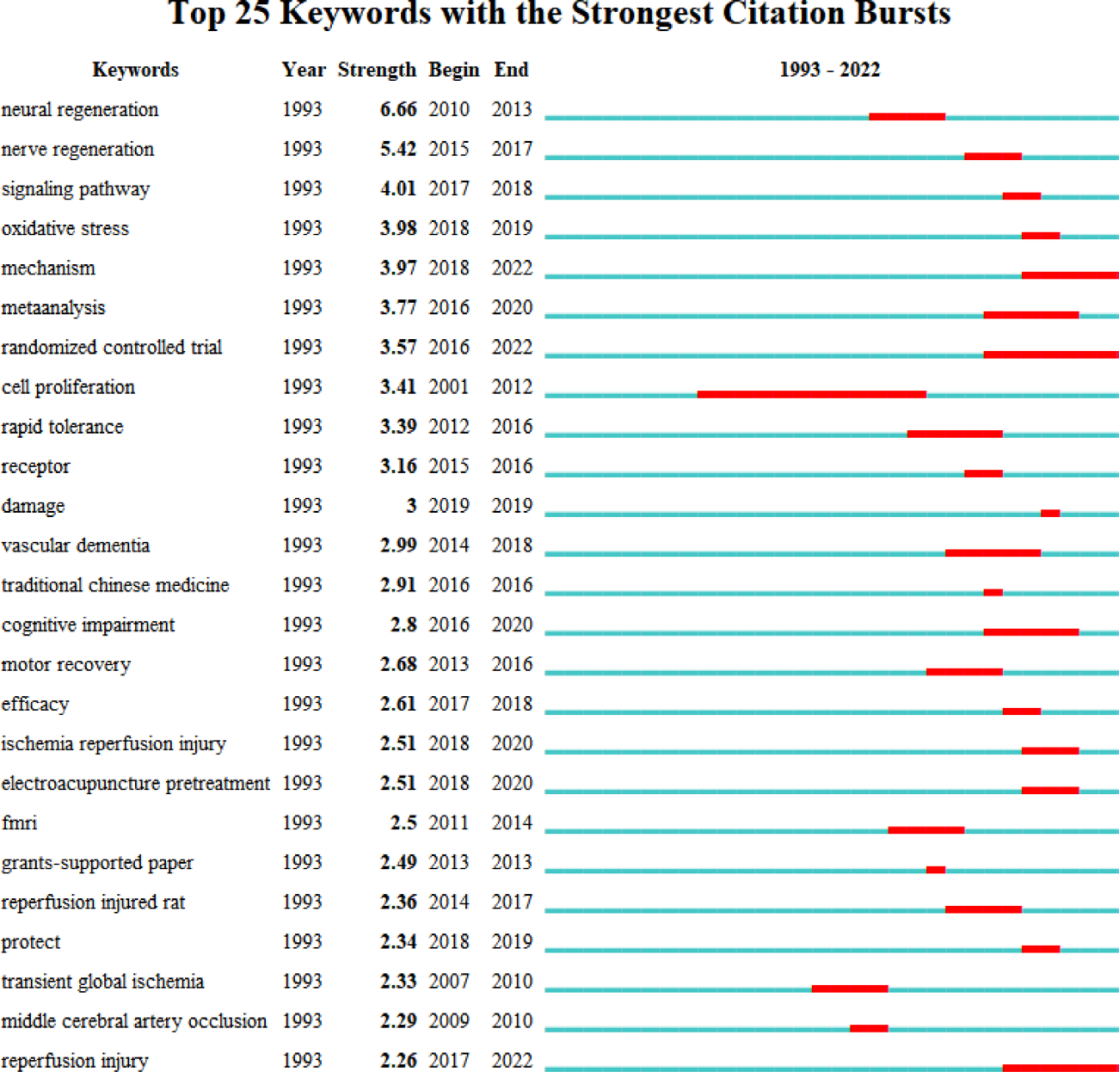
The first 25 keywords are highlighted.

Since 1993, the research related to acupuncture treatment of cerebral infarction has developed vigorously, among which the research on the mechanism of acupuncture treatment of cerebral infarction such as “neural regeneration” is the focus and main aspect. The emergence cycle of “cell proliferation” has the longest duration, “mechanism,” “randomized controlled trial” and “reperfusion injury” may become research hotspots in future studies, which deserve the attention of researchers (Fig. 7).

### 3.7. Keyword timeline analysis

**Figure 8.**
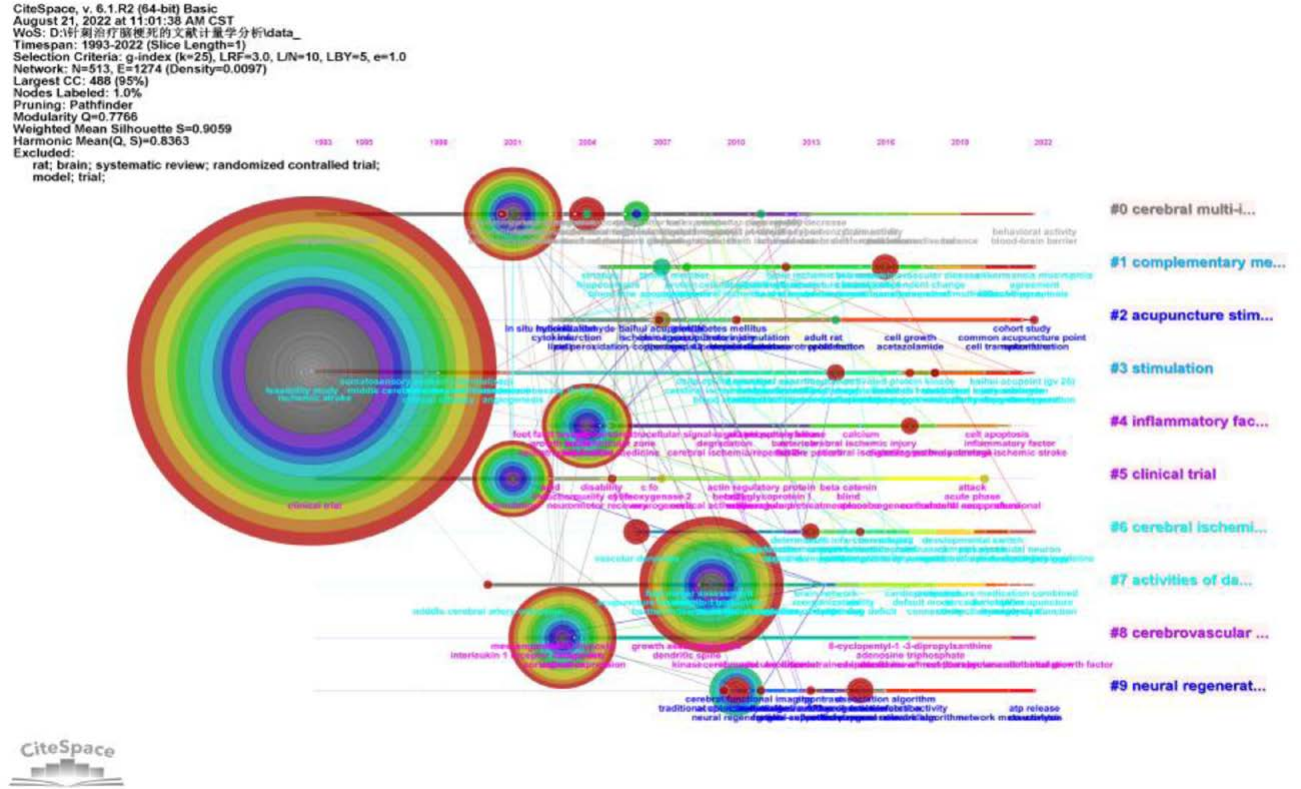
Keyword timeline.

According to the analysis of the keymap timeline map, in the first 10 clusters, the research heat of “acupuncture stimulation” is higher. Much of the recent research on acupuncture in the treatment of cerebral infarction has focused on “acupuncture stimulation,” “neural regeneration,” “cerebral multi- infarct,” “activities of daily living,” and “cerebrovascular circulation” is trending downward (Fig. 8).

### 3.8. Bibliometric analysis of co-cited references

**Figure 9.**
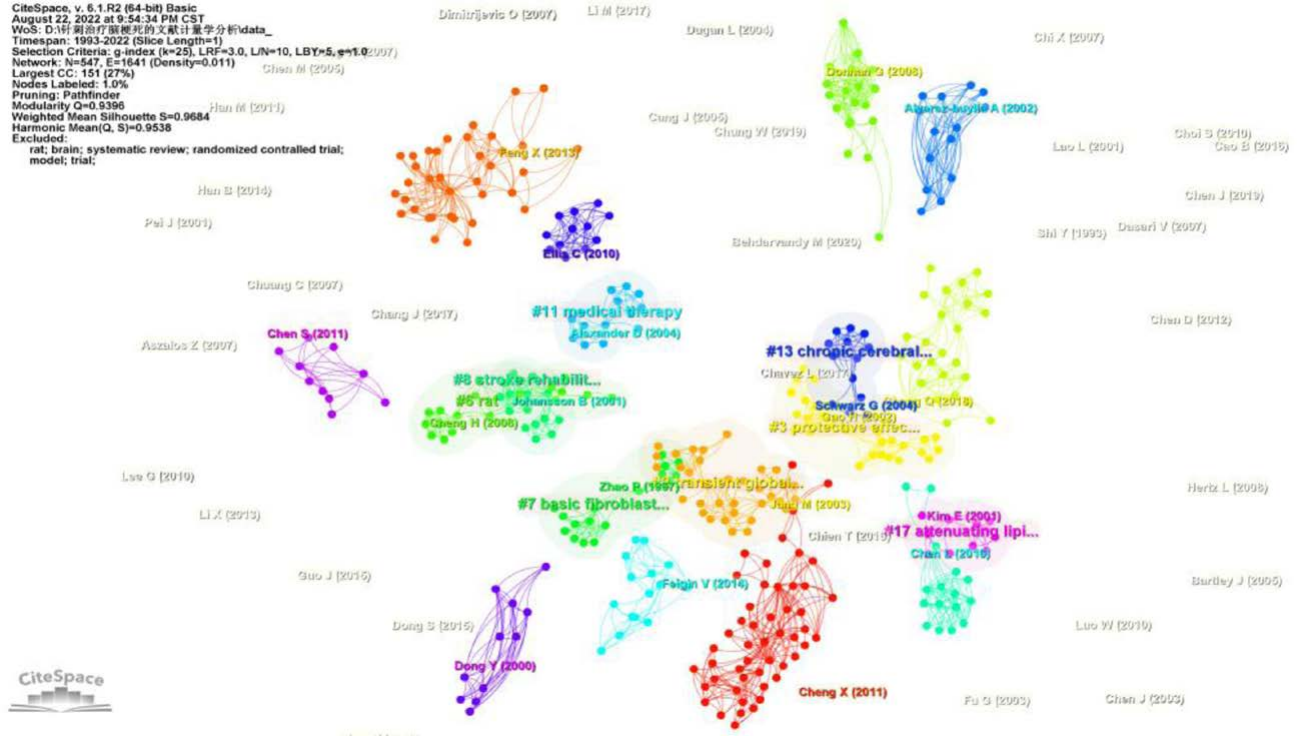
Network diagram of co-cited documents.

Of the 414 included papers, all of them have been cited at least once, with Gao H, 2002, *Acupuncture Electro*, 27, 45, and 7 citations (Table 5) (Fig. 9).

**Table 5:** Top 10 cited documents.

Citation counts	References	Doi	Cluster ID
7	Gao H, 2002, *Acupuncture Electro*, 27, 45	10.3727/036012902816026112	3
6	Kim E, 2001, *Neurosci Lett*, 297, 21	10.1016/S0304-3940(00)01656-6	17
6	Chavez L, 2017, *Int J Mol Sci*, 18, 0	10.3390/ijms18112270	22
5	Zhao P, 2000, *Acupuncture Electro*, 25, 101	10.3727/036012900816356163	69
4	Inoue I, 2002, *Neurosci Lett*, 333, 191	10.1016/S0304-3940(02)01032-7	3
4	Chang Q, 2018, *Neural Regen Res*, 13, 573	10.4103/1673-5374.230272	4
4	Chuang C, 2007, *Am J Chinese Med*, 35, 779	10.1142/S0192415X07005260	31
4	Wei G, 2000, *Acupuncture Electro*, 25, 81	10.3727/036012900816356208	2
4	Wu P, 2010, *Stroke*, 41, 0	10.1161/STROKEAHA.109.573576	50
4	Si Q, 1998, *Acupuncture Electro*, 23, 117	10.3727/036012998816356562	2

## 4. Discussion

### 4.1. Basic information

This is the first visual analysis of CiteSpace in the field of acupuncture treatment of cerebral infarction, aiming at revealing the basic situation, research hotspots, and frontiers in the field of acupuncture treatment of cerebral infarction. From the establishment of the WoSCC database to August 18, 2022, there are 414 publications related to acupuncture treatment of cerebral infarction, and the number of publications by year shows a stable growth trend. A total of 408 articles have been published in the top 5 countries in the field of research on acupuncture for cerebral infarction, 98.07% of the total number of publications. Among them, the country with the largest number of documents in this field is China. A total of 343 articles were published 82.85% of the total number of publications, about 4th to 5th of the total number of publications. It shows that China has great influence in research in this field and is in a dominant position. This also explains why the influential institutions in this research field all come from China. In this research field, the top 5 institutions are all Chinese higher education research institutions, with a total of 147 articles published, accounting for 35.51% of the total number of publications. Visualized analysis by countries and institutions provides researchers in this research field with a reference to the best partners. Top 10 authors published 180 articles, accounting for 43.48% of the total number of publications. These 10 authors are all from China, which is consistent with the visual analysis results of countries and institutions. Among them, the author with the highest number of published articles in the field of acupuncture for cerebral infarction is Chen L. He has published a total of 31 articles in the field, but in the co-cited visual analysis of the references. He is not the author with the largest number of citations in the literature, which shows that Chen L does not have close ties with authors from other countries or institutions and does not cooperate frequently enough. It is necessary to strengthen ties with authors from other countries or institutions and improve the quality of articles. In this area of research, the most cited author in the literature is Gao H’s article, but the total number of arguments is only 7 times. Moreover, the intermediary centrality of the top 10 articles with a total number of citations is <0.1, which indicates that there are no very influential articles in this research field. Researchers should pay more attention to the deep excavation of articles and the pursuit of article quality.

### 4.2. Hotspots and frontiers

Keyword analysis is usually used to study the research hotspots in this field and judge the development trend of this field. High-frequency keywords can be regarded as a hot topic for research in this field. High centrality keywords can be regarded as a topic with high influence in this field. In this study, high-center keywords and high-frequency keywords are basically the same (there are 6 groups of keywords in the top 10 keywords that are both high-center keywords and high-frequency keywords). Combined with the results of keyword clustering and co-occurrence, at present, the research hotspots and frontiers in this field mainly focus on the related contents of electroacupuncture in treating cerebral infarction and the mechanism of acupuncture in treating cerebral infarction. In particular, the mechanism of acupuncture treatment of cerebral infarction has attracted more attention from researchers.

Electroacupuncture for cerebral infarction electroacupuncture is an acupuncture method that outputs pulse radio waves from the electroacupuncture instrument to the millisecond needle on the basis of gas obtained after the ordinary millisecond needle is pierced into the acupoints, thus achieving the purpose of treating diseases.^[12]^ The stimulation parameters of electroacupuncture are waveform, frequency, intensity, and duration.^[13]^ The change of these parameters leads to the change of the pulse electricity output of the electroacupuncture instrument, so as to achieve the prevention and treatment effect of different diseases.^[14–16]^ Studies have shown that the most suitable duration for treating cerebral infarction is 30 minutes. This is the best time to reduce the damage of cerebral infarction nerve function and achieve analgesic effect. More than 30 minutes increases neurological impairment and mortality rate in patients with cerebral infarction.^[17]^ The most suitable stimulation frequency is 5 to 20 Hz. The most suitable stimulus intensity is 1.0 mA, within this optimal stimulus frequency and intensity. Electroacupuncture can effectively improve blood reperfusion in infarct areas, and this reperfusion effect is specific, thus reducing neurological damage and mortality rate.^[18]^ However, studies have also shown that when the stimulation frequency is between 15 and 30 Hz, electroacupuncture can maximize the integrity of the structure of astrocytes and play a role in protecting cerebral infarction from ischemic injury.^[19]^ Some scholars believe that, when the electroacupuncture strength is 3.0 mA, the activity of lactate dehydrogenase and succinate dehydrogenase can be increased thereby enhancing aerobic metabolism. At the same time, increases the activity of the enzyme Na^+^-K^+^-ATP, balancing the internal and external ions of brain cells, and reduces cellular edema.^[20]^ In addition, some articles suggest that the frequency and intensity of stimulation of electroacupuncture can change according to the changes of acupoints pierced by electroacupuncture.^[21]^ So far, the common waveforms used by electroacupuncture to treat cerebral infarction are continuous wave^[22]^ and dense wave.^[23]^ However, no scholars have made specific and systematic research on the waveform of electroacupuncture treatment of cerebral infarction. Therefore, in future research, scholars can pay more attention to this aspect and explore the waveform that is most suitable for treating cerebral infarction.

Mechanism of acupuncture in the treatment of cerebral infarction. According to the results of keyword analysis, it can be seen that the research hotspots of acupuncture in treating cerebral infarction mainly focus on reducing apoptosis of nerve cells and promoting nerve tissue remodeling. In the case of the former, many researchers^[24–26]^ started with gene expression related to the regulation of apoptosis in nerve cells. They think that acupuncture can regulate Bax, Cysteopathic asparaginase-3, and Bcl-2 expression of apoptosis-associated genes such as cIAP1. Thus, it plays a protective role in neural tissue. There are also many researchers who start with the relevant signaling pathways that regulate apoptosis. Tian and Wang^[27]^ concluded that acupuncture can inhibit apoptosis of cerebral infarction neurons by stimulating Notch3 signaling pathway and triggering corresponding protein expression. Sha et al^[28]^ believe that acupuncture can relieve neuronal inflammation by inhibiting miR-223/NLRP3 pathway, thus playing a protective role for nerves. Shi et al^[29]^ reported that acupuncture was able to activate the Wnt/β-catenin signaling pathway and up-regulate angiogenesis factor expression, thereby promoting angiogenesis after cerebral infarction. In promoting neural tissue remodeling, studies have shown that^[30,31]^ acupuncture can activate nerve growth factors and promote the proliferation of neural stem cells. It has also been studied that^[32,33]^ acupuncture can improve synaptic structure and function and promote synaptic repair after cerebral infarction. In addition, the inflammatory response plays a key role in ischemic neuronal injury. Acupuncture is able to inhibit the activation of microglia after ischemia, ^[21]^ reducing IL-6s in brain tissue, and TNF-α levels of inflammatory factors such as IL-1,^[34–36]^ so as to achieve the role of protecting neural tissue. Although the mechanism of acupuncture in treating ischemic stroke has been a hot topic of research. However, at present, the correlation between the mechanisms is not sufficient and there is no uniform standard for acupuncture methods and acupuncture points between various studies. Therefore, the research on the mechanism of action of acupuncture in treating cerebral infarction needs to be more standardized and systematic.

### 4.3. Advantages and limitations

This is the first time that CiteSpace has used acupuncture to treat cerebral infarction for bibliometric analysis. The literature search date is from the establishment of the database to August 18, 2022, and relevant articles in this field are included in the analysis as much as possible. According to the visualization results of CiteSpace, the past and present research of acupuncture treatment of cerebral infarction was observed, and the research hotspots and frontiers in this field were explored in the future. However, because we only searched WoSCC and set a large number of keywords, we may miss some articles with more analytical significance, which is the limitation of this research.

## 5. Conclusion

According to the visual map analysis of CiteSpace, this study shows that in the field of acupuncture treatment of cerebral infarction, the cooperation among various institutions in China is the closest, and the cooperation among various countries is less and more scattered. At present, the research hotspots and frontiers in this field mainly focus on the related contents of electroacupuncture in treating cerebral infarction and the mechanism of acupuncture in treating cerebral infarction. Therefore, more attention should be paid to the study of electroacupuncture for cerebral infarction and the mechanism of acupuncture for cerebral infarction.

## Acknowledgments

Thanks to all authors for their contributions to this article.

## Author contributions

Conceptualization: Yutong Han, Lei Shi.

Data curation: Yutong Han, Chang Liu.

Methodology: Yutong Han.

Visualization: Yutong Han, Huasong Gao.

Writing—review & editing: Yutong Han, Chang Liu, Lei Shi.

Investigation: Xinming Yang, Jiaxiao Zhou, Weiping Shi.

Software: Huasong Gao, Huixue Zhang, Dawei Ran.

Formal analysis: Lei Shi.

Writing—original draft: Lei Shi.

## References

[1] Feigin V Brainin M. Reducing the burden of stroke: opportunities and mechanisms. Int J Stroke. 2019;14:761–2. 31496437 10.1177/1747493019874718

[2] Du X Liu Q Li Q. Prognostic value of cerebral infarction coefficient in patients with massive cerebral infarction. Clin Neurol Neurosurg. 2020;196:106009. 32554235 10.1016/j.clineuro.2020.106009

[3] Li L Zhang H Meng SQ. An updated meta-analysis of the efficacy and safety of acupuncture treatment for cerebral infarction. PLoS One. 2014;9:e114057. 25438041 10.1371/journal.pone.0114057 PMC4250085

[4] Wang Y Xing J Li Y. Effect and safety of acupuncture on cerebrovascular reserve in patients with acute cerebral infarction: a protocol for systematic review and meta-analysis. Medicine (Baltimore). 2021;100:e26636. 34260557 10.1097/MD.0000000000026636 PMC8284725

[5] Wang LX Li WH He F. Efficacy and safety of electroacupuncture in the treatment of cerebral infarction: systematic review and meta-analysis. Appl Bionics Biomech. 2022;2022:1350501. 35800118 10.1155/2022/1350501 PMC9256421

[6] Chavez LM Huang SS MacDonald I. Mechanisms of acupuncture therapy in ischemic stroke rehabilitation: a literature review of basic studies. Int J Mol Sci. 2017;18:2270. 29143805 10.3390/ijms18112270 PMC5713240

[7] Yue J Liu M Li J. Acupuncture for the treatment of hiccups following stroke: a systematic review and meta-analysis. Acupunct Med. 2017;35:2–8. 27286862 10.1136/acupmed-2015-011024

[8] Sun Z Yu N Yue J. Acupuncture for urinary incontinence after stroke: a protocol for systematic review. BMJ Open. 2016;6:e008062. 10.1136/bmjopen-2015-008062 PMC4769427 26908510

[9] Chen C Hu Z Liu S. Emerging trends in regenerative medicine: a scientometric analysis in CiteSpace. Expert Opin Biol Ther. 2012;12:593–608. 22443895 10.1517/14712598.2012.674507

[10] Zhong Y Cao J Lu H. Publication trends in rehabilitative effects of acupuncture: a visual analysis of the literature. Evid Based Complement Alternat Med. 2022;2022:7705256. 35449821 10.1155/2022/7705256 PMC9017514

[11] Zhang J Zhang Y Hu L. Global trends and performances of magnetic resonance imaging studies on acupuncture: a bibliometric analysis. Front Neurosci. 2021;14:620555. 33551731 10.3389/fnins.2020.620555 PMC7854454

[12] Liu B Wu J Yan S. Electroacupuncture vs prucalopride for severe chronic constipation: a multicenter, randomized, controlled, noninferiority trial. Am J Gastroenterol. 2021;116:1024–35. 33273258 10.14309/ajg.0000000000001050

[13] Liu Z Liu Y Xu H. Effect of electroacupuncture on urinary leakage among women with stress urinary incontinence: a randomized clinical trial. JAMA. 2017;317:2493–501. 28655016 10.1001/jama.2017.7220 PMC5815072

[14] Yu X. Potential value of electroacupuncture in the treatment of gastrointestinal symptoms of COVID-19. Inflamm Bowel Dis. 2022;28:e21. 34424332 10.1093/ibd/izab223 PMC8499809

[15] Lee B Kim BK Kim M. Electroacupuncture for treating cancer-related insomnia: a multicenter, assessor-blinded, randomized controlled, pilot clinical trial. BMC Complement Med Ther. 2022;22:77. 35303841 10.1186/s12906-022-03561-w PMC8932204

[16] Li C Qu Z Liu J. Effect of electroacupuncture on the intestinal microflora in rats with stress urinary incontinence. Front Endocrinol (Lausanne). 2022;13:860100. 35992152 10.3389/fendo.2022.860100 PMC9390059

[17] Zhou F Guo J Cheng J. Effect of electroacupuncture on rat ischemic brain injury: importance of stimulation duration. Evid Based Complement Alternat Med. 2013;2013:878521. 23737851 10.1155/2013/878521 PMC3666426

[18] Zhou F Guo J Cheng J. Electroacupuncture increased cerebral blood flow and reduced ischemic brain injury: dependence on stimulation intensity and frequency. J Appl Physiol (1985). 2011;111:1877–87. 21836043 10.1152/japplphysiol.00313.2011 PMC3233896

[19] Xiao Y Wu X Deng X. Optimal electroacupuncture frequency for maintaining astrocyte structural integrity in cerebral ischemia. Neural Regen Res. 2013;8:1122–31. 25206406 10.3969/j.issn.1673-5374.2013.12.007 PMC4145895

[20] Tian WQ Peng YG Cui SY. Effects of electroacupuncture of different intensities on energy metabolism of mitochondria of brain cells in rats with cerebral ischemia-reperfusion injury. Chin J Integr Med. 2015;21:618–23. 24002710 10.1007/s11655-013-1512-9

[21] Liao SL Lin YW Hsieh CL. Neuronal regeneration after electroacupuncture treatment in ischemia-reperfusion-injured cerebral infarction rats. Biomed Res Int. 2017;2017:3178014. 28913350 10.1155/2017/3178014 PMC5587926

[22] Shi L Cao HM Li Y. Electroacupuncture improves neurovascular unit reconstruction by promoting collateral circulation and angiogenesis. Neural Regen Res. 2017;12:2000–6. 29323038 10.4103/1673-5374.221156 PMC5784347

[23] Zhou F Guo J Cheng J. Electroacupuncture and brain protection against cerebral ischemia: specific effects of acupoints. Evid Based Complement Alternat Med. 2013;2013:804397. 23737846 10.1155/2013/804397 PMC3666307

[24] Wang SJ Omori N Li F. Potentiation of Akt and suppression of caspase-9 activations by electroacupuncture after transient middle cerebral artery occlusion in rats. Neurosci Lett. 2002;331:115–8. 12361854 10.1016/s0304-3940(02)00866-2

[25] Xu XY Fang Q Huang W. Effect of electroacupuncture on neurological deficit and activity of clock and Bmal1 in cerebral ischemic rats. Curr Med Sci. 2020;40:1128–36. 33428141 10.1007/s11596-020-2295-9

[26] Tang Q Ye T Liang R. Scalp acupuncture and treadmill training inhibits neuronal apoptosis through activating cIAP1 in cerebral ischemia rats. Evid Based Complement Alternat Med. 2021;2021:1418616. 34804173 10.1155/2021/1418616 PMC8604578

[27] Tian R Wang S. Electroacupuncture reduced apoptosis of hippocampal neurons in mice with cerebral infarction by regulating the Notch3 signaling pathway. J Mol Neurosci. 2019;67:456–66. 30726543 10.1007/s12031-018-1253-5

[28] Sha R Zhang B Han X. Electroacupuncture alleviates ischemic brain injury by inhibiting the miR-223/NLRP3 pathway. Med Sci Monit. 2019;25:4723–33. 31237865 10.12659/MSM.917213 PMC6607941

[29] Shi S Wang M Liu X. Scalp electroacupuncture promotes angiogenesis after stroke in rats by activation of Wnt/*β*-catenin signal pathway. Evid Based Complement Alternat Med. 2022;2022:1649605. 35321503 10.1155/2022/1649605 PMC8938052

[30] Kim YR Kim HN Ahn SM. Electroacupuncture promotes post-stroke functional recovery via enhancing endogenous neurogenesis in mouse focal cerebral ischemia. PLoS One. 2014;9:e90000. 24587178 10.1371/journal.pone.0090000 PMC3933702

[31] Luo D Fan X Ma C. A study on the effect of neurogenesis and regulation of GSK3β/PP2A expression in acupuncture treatment of neural functional damage caused by focal ischemia in MCAO rats. Evid Based Complement Alternat Med. 2014;2014:962343. 25120577 10.1155/2014/962343 PMC4120913

[32] Yi W Xu NG Wang GB. Experimental study on effects of electro-acupuncture in improving synaptic plasticity in focal cerebral ischemia rats. Zhongguo Zhong Xi Yi Jie He Za Zhi. 2006;26:710–4. 16970094

[33] Shen MH Tang QQ Li ZR. Effect of electroacupuncture on hippocampal LTP in Alzheimer’ s disease rats induced by Abeta(25-35). Zhen Ci Yan Jiu. 2010;35:3–7. 20458898

[34] Chen Y Huang W Li Z. The effect of acupuncture on the expression of inflammatory factors TNF-α, IL-6, IL-1 and CRP in cerebral infarction: a protocol of systematic review and meta-analysis. Medicine (Baltimore). 2019;98:e15408. 31192907 10.1097/MD.0000000000015408 PMC6587630

[35] Wong V Cheuk DK Chu V. Acupuncture for hypoxic ischemic encephalopathy in neonates. Cochrane Database Syst Rev. 2013;2013:CD007968. 23440822 10.1002/14651858.CD007968.pub2 PMC6885036

[36] Lan L Tao J Chen A. Electroacupuncture exerts anti-inflammatory effects in cerebral ischemia-reperfusion injured rats via suppression of the TLR4/NF-κB pathway. Int J Mol Med. 2013;31:75–80. 23165960 10.3892/ijmm.2012.1184

